# EZH2 mediates lidamycin-induced cellular senescence through regulating p21 expression in human colon cancer cells

**DOI:** 10.1038/cddis.2016.383

**Published:** 2016-11-24

**Authors:** Ming-Quan Sha, Xiao-Li Zhao, Liang Li, Li-Hui Li, Yi Li, Tian-Geng Dong, Wei-Xin Niu, Li-Jun Jia, Rong-Guang Shao, Yong-Su Zhen, Zhen Wang

**Affiliations:** 1 Department of Biochemistry, Institute of Medicinal Biotechnology, Chinese Academy of Medical Sciences and Peking Union Medical College, Beijing, China; 2 Cancer Institute, Fudan University Shanghai Cancer Center; Department of Oncology, Shanghai Medical College, Fudan University, Shanghai, China; 3 Department of General Surgery, Zhongshan Hospital, Fudan University, Shanghai, China

## Abstract

Lidamycin (LDM) is a novel member of the enediyne antibiotics identified in China with potent antitumor activity. However, it remains unclear whether LDM has potential molecular targets that may affect its antitumor activity. Enhancer of zeste homolog 2 (EZH2) functions as a histone lysine methyltransferase and mediates trimethylation on histone 3 lysine 27 (H3K27me3). High EZH2 level is found to be positively correlated with the aggressiveness, metastasis and poor prognosis of cancer. Here, we aim to study the role of EZH2 in LDM-induced senescence, as well as in the cytotoxicity of LDM in human colon cancer cells. LDM is found to be relatively more potent in inhibiting the colon cancer cells harboring high EZH2 level and induces irreversible cellular senescence at IC_50_ dose range, as evidenced by senescence-associated *β*-galactosidase staining, cell cycle arrest and molecular changes of senescence regulators including p21 in HCT116 and SW620 cells. More importantly, LDM is found to markedly inhibit EZH2 expression at both protein and mRNA levels upon the induction of p21 and cellular senescence. LDM also selectively inhibits EZH2 expression as compared with other histone lysine methyltransferases. Knockdown of p21 with siRNAs abolishes LDM-induced senescence, whereas EZH2 knockdown markedly increases p21 expression and causes senescent phenotype. Enrichment of both EZH2 and H3K27me3 levels in the p21 promoter region is reduced by LDM. Moreover, EZH2 overexpression reduces cellular senescence, p21 expression and DNA damage response upon LDM exposure. LDM also demonstrates potent antitumor efficacy in xenografted animal models. Collectively, our work provides first demonstration that EZH2 may mediate, at least partially, the senescence-inducing effects of LDM by regulating p21 expression and DNA damage effect. Thus, EZH2 may serve as a potential target and biomarker to indicate the clinical efficacy of the potent enediyne antitumor drug.

Polycomb repressor complex 2 (PRC2) includes three core components: enhancer of zeste homolog 2 (EZH2), SUZ12 and EED, and functions as a pivotal regulator of cell growth, survival and differentiation.^1^ As a histone lysine methyltransferase, EZH2 is a major catalytic component of PRC2 and mediates trimethylation on histone 3 lysine 27 (H3K27me3), resulting in the silencing of mostly tumor-suppressing genes.^2^ High EZH2 levels are found in many human malignant solid tumors and positively correlate with the initiation, as well as drug resistance of cancer, indicating poor prognosis. In addition, EZH2 activating mutations are found to occur in some types of lymphoma, further rendering EZH2 as an appealing target in cancer therapy.^3, 4, 5, 6^

Enediyne antibiotics have been shown to have potent antitumor activity because of their unique ability to damage DNA of tumor cells by inducing sequence-specific single-strand and/or double-strand breaks. Lidamycin (LDM, originally named as C-1027), isolated from a *strptomycesglobisporus* C-1027 strain and currently under phase II clinical trial in China, is one of most potent members of the enediyne family.^7^ LDM consists of an apoprotein and a chromophore, with the former serving as protecting protein and the latter as active component to damage DNA.^8^ It has been proved that LDM-induced DNA damages are mimetic that of ionizing radiation.^9^ Previous studies indicate LDM is highly toxic to a variety of tumor cell lines both *in vitro* and *in vivo*. The potent cytotoxicity of LDM also makes it a promising immunotoxin in targeted cancer therapy.^10^ Our previous studies have suggested that LDM induces apoptosis in both p53-dependent and -independent manner, as well as caspases-independent manner, based on different dosage range in cancer cells.^11, 12^ Moreover, LDM is shown to have strong inhibitory effect on tumor metastasis, angiogenesis and tumor-initiating cells.^13^ Although the potent antitumor effects of LDM are known to be closely related to its specific chemical structure, it remains to be elucidated whether the agent has potential molecular targets that may affect its antitumor activity.

Cellular senescence results in irreversible growth arrest in malignant cancer cells, thus becoming a vital mechanism of tumor suppression. Previous reports have showed that EZH2 is involved in the regulation of senescence in human progenitor cells, as well as mouse embryonic fibroblasts (MEFs) through inhibition of *INK4b (p16)* and *INK4a (p15)* locus.^14, 15^ Also, EZH2 depletion causes senescence of melanoma cells.^16^ As LDM is found to induce senescence in tumor cells with yet undefined mechanism,^17^ we speculate that EZH2 may be involved in the regulation of LDM-induced senescence. In this work, we used human colon cancer cells to check the role of EZH2 in the antitumor effect of LDM, and found that EZH2 mediates the cellular senescence through regulating p21 expression.

## Results

### EZH2 is highly expressed in clinical colon cancer tissues and positively correlates with the disease grades

**Figure 1:**
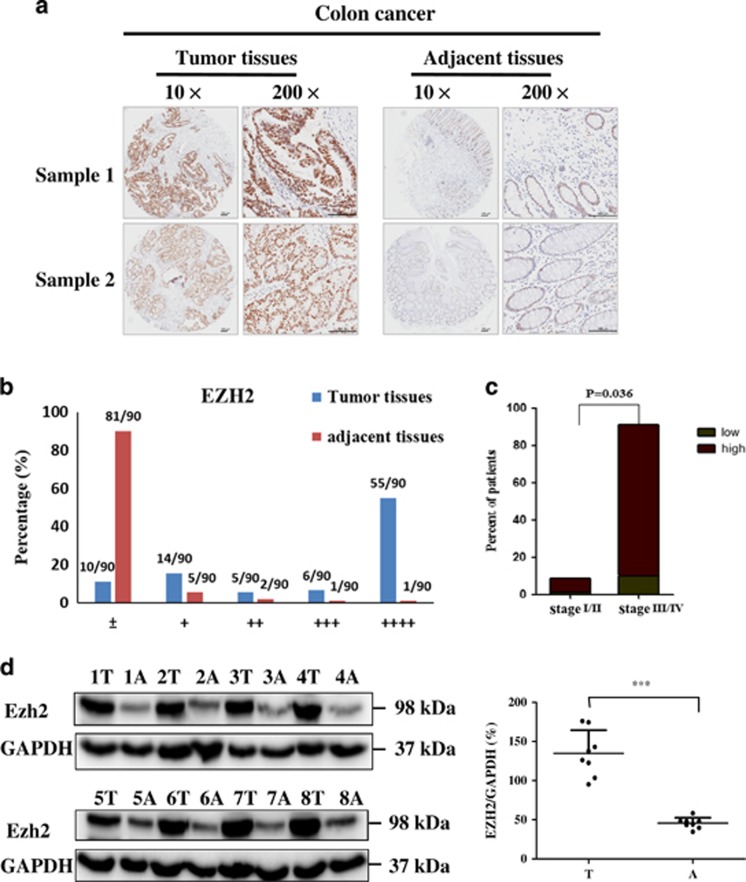
Expression of EZH2 in human colon cancer tissues. (**a**) IHC staining of EZH2 in both tumoral (left) and adjacent (right) tissues from two representative samples on the tissue array. Different magnification from the same sample was shown (scale bar: 100 *μ*m). (**b**) Based on the EZH2 staining intensity, five groups were classified and plotted from the weakest (±) to the strongest (++++) in both tumoral and adjacent tissues on the tissue array (*n*=90), as described in Materials and methods section. (**c**) High EZH2 expression was correlated with TNM stage (III/IV *versus* I/II) (*P*<0.05) in the samples according to their clinicopathologic features listed in Supplementary Table 1. (**d**) IB analysis of EZH2 expression in eight pairs of colon cancer and adjacent normal tissues from patients. GAPDH was used as a loading control. Quantification of the protein expression in the tumor (T) and adjacent tissues (A) was analyzed on the right (*n*=8) (***P<0.001)

We first performed immunohistochemistry (IHC) staining to analyze EZH2 expression in human colon cancer tissue array containing 90 primary clinical samples with both tumoral and adjacent normal tissues. As shown in Figure 1a, EZH2 was highly expressed in the nuclei of illustrated tumor samples as compared with the same adjacent tissues in two representative staining samples. Based on the staining intensity, we classified the samples into five groups with increasing intensity, from marginal (±) to weak (+) and the strongest (++++). In all, 90% of the normal tissues demonstrated marginal expression, whereas 73.3% tumor tissues showed strong staining (falling into group ++ to ++++) (Figure 1b). The strongest EZH2 expression was seen in 55 of 90 tumor samples (61.1%), but in only 1 of 90 adjacent tissues (1.1%). Further analysis showed that EZH2 expression positively correlated with tumor pathological differentiation grades, with much higher expression in grades III/IV (more poorly differentiated) than that of grades I/II (91.1% *versus* 8.9%, respectively, *P*<0.05) (Figure 1c), whereas being independent of age, tumor size and TNM stage (Supplementary Table 1).

We further tested EZH2 expression in eight paired samples (colon cancer *versus* adjacent samples) from cancer patients in Zhongshan hospital with immunoblotting (IB) analysis. In Figure 1d, EZH2 levels in tumor tissues were consistently and significantly higher than that of adjacent ones. Thus, our results suggest that high EZH2 expression may be involved in the progression of colon cancer, and serve as a potential biomarker, as well as treatment target for human colon cancer patients.

### LDM exerts potent growth inhibitory effect toward colon cancer largely through inducing senescence at IC_50_ dose range

**Figure 2:**
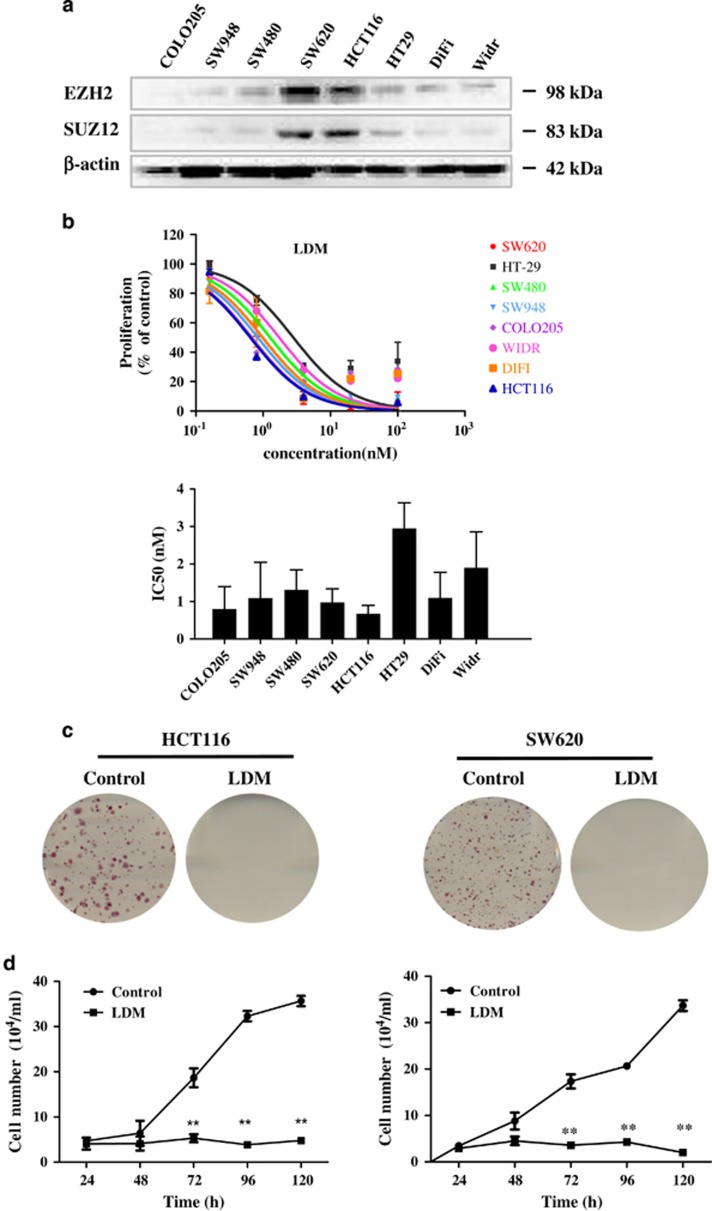
Expression of EZH2 family members in human colon cancer cells and effects of LDM on the cells. (**a**) Expression of EZH2 and SUZ12 proteins in different human colon cancer cells were detected by IB analysis. (**b**) The cells were seeded in 96-well plates for 24 h, and then treated with different concentrations of LDM for another 48 h before subjected to MTT assay. Upper panel shows the inhibitory of proliferation curve and lower panel shows the IC_50_ value of each cell line determined from three independent experiments. (**c**) Representative result of LDM (0.5 nM) on the colony formation from three independent experiments for 2 weeks. (**d**) HCT116 and SW620 cells were treated with or without LDM (0.5 nM) for 24–120 h. Cellular numbers were counted at the indicated time points with a Countess Cell Counter. **P<0.01 *versus* control at respective time. The data were expressed as the means±S.D. of three independent experiments

To evaluate whether there may be any correlation between EZH2 expression and LDM cytotoxicity, we compared the basal EZH2 protein expression in a panel of human colon cancer cell lines and IC_50_ of LDM. EZH2 expression was relatively high in HCT116 and SW620 cell lines compared with other cell lines (Figure 2a). Moreover, a parallel high expression of SUZ12, a member of PRC2 core complex, was also observed. We then compared the proliferation inhibitory effect of LDM upon 48-h treatment across these cell lines, and found that LDM was relatively more potent in HCT116 and SW620 cells (with IC_50_ being 0.67 and 0.97 nM, respectively) among the tested cells, indicating that cancer cells with higher EZH2 expression were more susceptible to LDM-induced cytotoxicity (Figure 2b).

**Figure 3:**
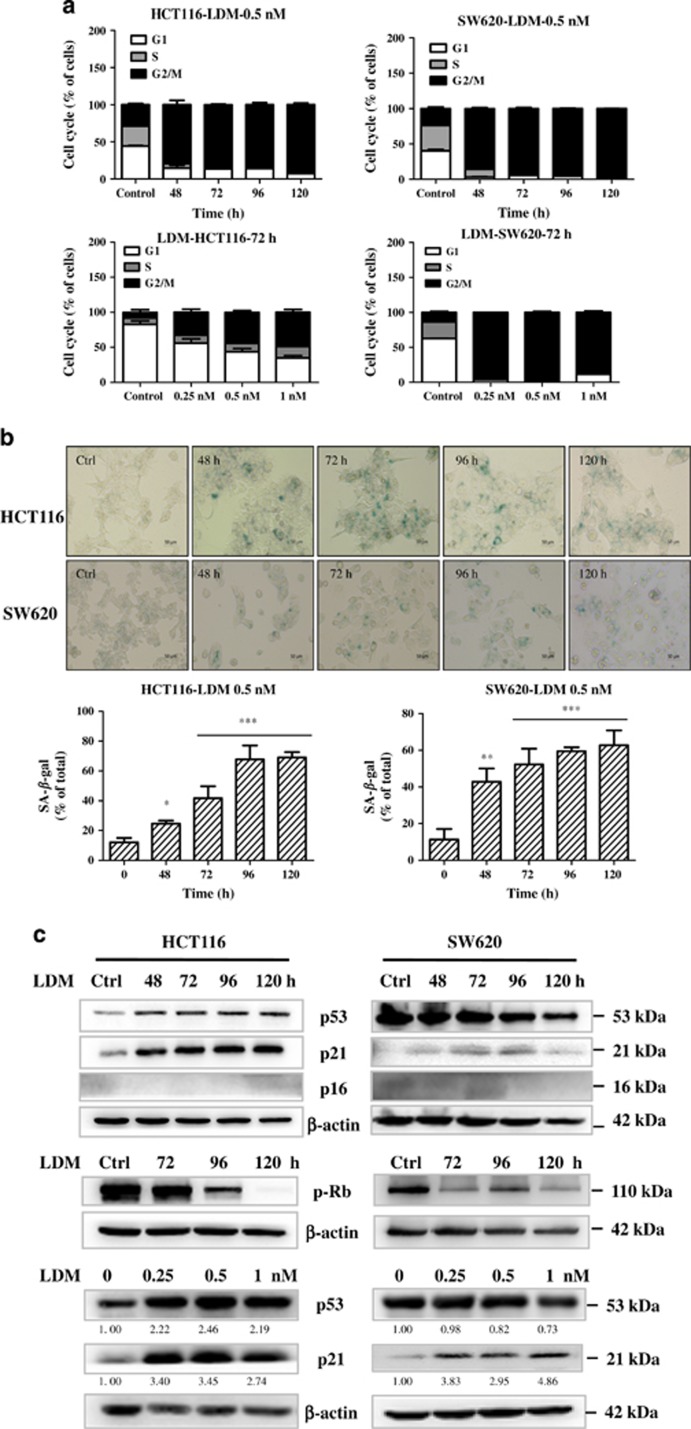
LDM induced growth arrest and senescence in the colon cancer cells. (**a**) Cell cycle distribution upon LDM treatment in both time- and dose-dependent manner as indicated. The data were representative from three independent experiments. (**b**) LDM-induced senescence in the cells. The cells were treated with 0.5 nM LDM for the indicated time and analyzed with SA-*β*-Gal staining and quantified (× 20). The data were expressed as the means±S.D. of three independent experiments (*P<0.05, **P<0.01, ***P<0.001 *versus* control). (**c**) LDM affected the molecular expression of proteins involved in senescence regulation. The cells were treated either with 0.5 nM LDM for the indicated time, or with different doses of the drug for 72 h, and then subjected to IB analysis using respective antibodies. *β*-Actin served as a loading control. The relative optical density for each band was quantified, normalized and labeled under each lane

To further determine the effect of LDM, growth inhibition curve and colony formation were assayed. As shown in Figure 2d, LDM markedly inhibited the growth curve of both HCT116 and SW620 cells, ranging from 86 to 94% inhibition on day 5. Longer exposure to LDM caused very few colonies formed (Figure 2c). Our and other's previous studies have shown that LDM-induced cytotoxicity may result from apoptosis and/or cell cycle arrest.^12, 18, 19^ We next investigated the induction of apoptosis and cell cycle distribution changes upon 0.5 nM LDM treatment for 48–120 h with FACS analysis, and found that LDM induced 16.5% and 24.7% apoptosis in HCT116 cells, and 16.9% and 17.5% apoptosis in SW620 cells after treatment for 72 h and 96 h, respectively (Supplementary Figure S1a). Furthermore, LDM induced more significant G2/M phase arrest in both time- and dose-dependent manner, reaching nearly 90% G2/M phase after drug treatment for 120 h (Figure 3a).

As cell cycle arrest is one of the main features of cellular senescence, we next determined whether LDM-induced senescence in both HCT116 and SW620 cells using the well-accepted senescence-associated *β*-galactosidase (SA-*β*-Gal) staining method.^20^ As shown in Figure 3b, significant induction of senescent phenotype was observed time dependently after 0.5 nM LDM treatment for 48–120 h, with the ratio of senescent cells reaching over 60% in both cells on day 5. Lower dose (0.25 nM) LDM also caused significant senescence (Supplementary Figure S1b). Thus, our data suggest that LDM at IC_50_ level possesses potent growth inhibitory effect toward colon cancer cells largely through inducing senescence.

### P21 signaling pathway is involved in LDM-caused senescence

To further address the underlying mechanisms of LDM-caused senescence, we first checked whether p53-p21 and p16-pRb signaling pathways may be involved, both of which are well-known regulators during cellular senescence.^21^ Consistent with our previous work,^12^ p53 is found to be increased after LDM treatment for 48–120 h in HCT116 cells harboring wild-type p53 (Figure 3c). Meanwhile, p53 downstream target p21 was increased. Although little changes of p53 expression were seen in SW620 cells carrying mutant p53, p21 level remained to be enhanced. The same tendency was also found dose dependently (Figure 3c). To further prove p21 induction is independent of p53, we tested the effect of LDM in HCT116 p53−/− cells, and similarly, p21 expression is increased (Supplementary Figures S2a–c). In addition, little changes of p16 level were observed, whereas pRb (Ser807/811) levels were decreased time dependently in both cells (Figure 3c, middle panel). As p21 induction can also lead to the inhibition of Rb phosphorylation by inhibiting CDK2/cyclin E activity,^22^ we conclude that p21-mediated signaling pathway is involved in the LDM-caused senescence.

### LDM decreases EZH2 expression at both protein and mRNA levels upon p21 induction

**Figure 4:**
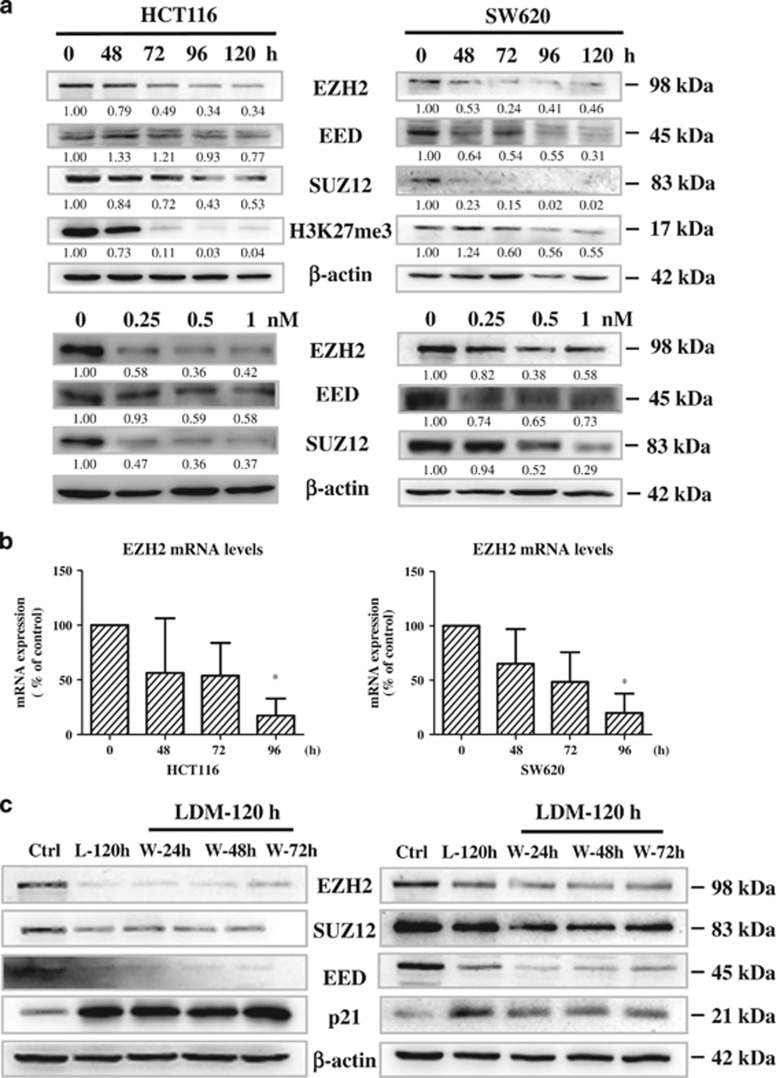
LDM reduced the expression levels of EZH2 family members. (**a**) Time- and dose-dependent effect of LDM in HCT116 and SW620 cells. The cells were treated with 0.5 nM LDM for the indicated time or 72 h at the indicated doses. *β*-Actin served as loading control. The relative optical density for each band was quantified, normalized and labeled under each lane. (**b**) The cells were treated with 0.5 nM LDM as indicated, harvested and subjected to qRT-PCR analysis. The data were expressed as the means±S.D. of four independent experiments. *P<0.05 *versus* control. (**c**) The cells were treated with 0.5 nM LDM for 24, 48 and 72 h, followed by washing with PBS and culture in drug-free medium until 120 h (labeled as W-24 h, W-48 h, W-72 h, respectively), and then harvested and subjected to IB analysis. Long exposure to LDM for 120 h without washing was labeled as L-120 h and loaded simultaneously

Given that LDM-induced p21 expression was independent of p53, and p21 is one of epigenetic targets of EZH2,^16, 23, 24^ we next sought to understand whether EZH2 may be involved in p21 regulation upon the senescence induction. We first checked the expression level of EZH2 after LDM treatment. In Figure 4a, significant reduction of EZH2 protein expression, along with two other PRC2 family members EED and SUZ12, was observed after LDM treatment for various time in the colon cancer cells. H3K27me3, a molecular target manifesting EZH2 methyltransferase activity, was reduced as well. Similar reductions of EZH2, EED and SUZ12 protein levels were observed by LDM treatment at various doses for 72 h (Figure 4a, lower panel). Despite colon cancer, we also checked other cancer cell lines from different tissues, and observed similar tendency for the PRC2 family members upon LDM treatment (Supplementary Figure S3a). To test whether the reduction of EZH2 protein level may result from increased proteasome degradation, we added proteasome inhibitor MG132 upon LDM treatment and found little changes of EZH2 expression, indicating that the regulation of EZH2 may occur at mRNA level (Supplementary Figure S3b). Further RT-PCR analysis confirmed significant reduction of *EZH2* mRNA upon LDM treatment time dependently (Figure 4b). To check the specificity of LDM toward EZH2, we examined whether LDM may affect gene expression of other related histone lysine methyltransferases, including EZH1, SUV39H1 and G9a. EZH1 is homologous to EZH2 and forms similar PRC2 complex with yet distinct functions.^25^ SUV39H1 and G9a are found to mediate H3K9 methylation, another form of repressive histone modification.^26^ As shown in Supplementary Figure S3c, although fluctuant changes were observed at different time points, LDM in general did not take significant effect on the mRNA levels of *EZH1*, *SUV39H1* and *G9a* as analyzed by RT-PCR. Meanwhile, H3K9me3, a target manifesting SUV39H1 activity, remained unchanged (Supplementary Figure S3d). This suggests that LDM specifically inhibits *EZH2* mRNA expression.

We further tested whether the expression changes of EZH2 family proteins and p21 were reversible after removal of the drug. In Figure 4c, after treatment with LDM for 24–72 h, the medium was replaced with drug-free medium, and the decrease of EZH2 family members, as well as increase of p21 were found to last until 120 h free of the drug exposure, indicating irreversible change of the molecular expression levels. Supportively, the inverted expression of EZH2 and p21 was confirmed in HCT116 p53−/− cells (Supplementary Figure S2c). Therefore, our data suggest that LDM reduces EZH2 while inducing p21 protein expression upon senescence development in the cancer cells.

### EZH2 negatively regulates p21 and mediates LDM-induced senescence

**Figure 5:**
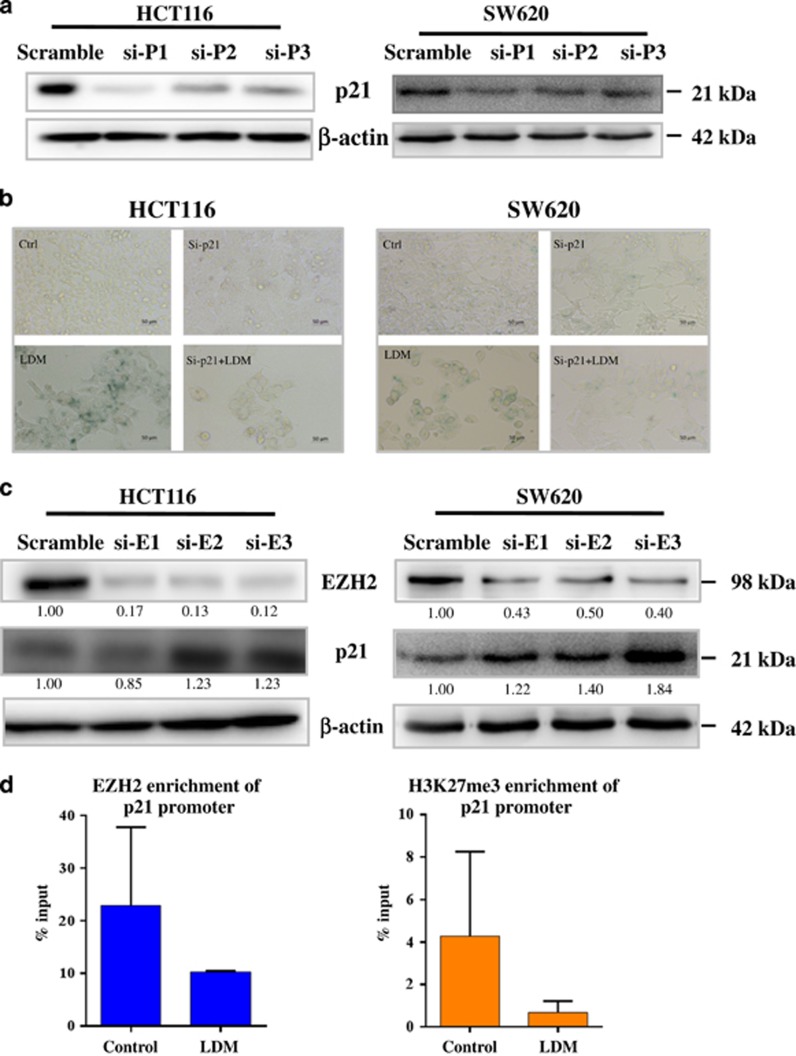
EZH2 negatively regulated p21 expression upon LDM exposure. (**a**) The cells were transfected with three siRNAs targeting p21 for 48 h, followed by IB analysis. (**b**) Depletion of p21 abolished LDM-induced senescence. The cells were transfected with siRNA targeting p21 (1#) for 24 h, and then subjected to 0.5 nM LDM treatment for 72 h before SA-*β*-Gal staining (× 20). Scale bar: 50 *μ*m. (**c**) EZH2 Knockdown induced p21 expression. The cells were transfected with three siRNAs targeting EZH2 for 48 h, followed by IB analysis. The relative optical density for each band was quantified, normalized and labeled under each lane. (**d**) LDM reduced the enrichment of EZH2 and H3K27me3 levels in *p21* promoter region analyzed by ChIP assay. SW620 cells were treated with LDM for 72 h before subjected to ChIP assay as described in Materials and methods section. The data were expressed as the means±S.D. of three independent experiments

We next analyzed the mechanistic regulation involved in p21 expression by EZH2. We first verified the role of p21 in the senescence by knocking down *p21* expression with siRNAs. In Figure 5a, three different siRNAs all markedly blocked p21 expression in both HCT116 and SW620 cells, with the first one being most efficient. We then used this siRNA to knockdown *p21* before further LDM treatment, and found that reduction of p21 expression significantly blocked LDM-induced senescence (Figure 5b), confirming that p21 mediated the senescence.

We then checked whether *EZH2* knockdown may affect p21 expression, as well as senescent phenotype. As shown in Figure 5c, three siRNAs caused apparent reduction of EZH2 expression in both cells, and all the siRNAs significantly increased p21 expression in SW620 cells, whereas two of them (2# and 3#) induced marked p21 expression in HCT116 cells. Although si-EZH2 (1#) failed to increase p21 expression in HCT116 cells for unknown reason, this result confirms that EZH2 negatively regulated p21 expression. Furthermore, *EZH2* knockdown with siRNA (3#) caused senescent phenotype (Supplementary Figure S4a), indicating that EZH2 may mediate LDM-induced senescence as well. Supportively, senescent phenotypes were rarely seen in Widr and SW948 cells upon LDM treatment (Supplementary Figure S4b), both of which carry relatively lower level of endogenous EZH2 (Figure 2a).

**Figure 6:**
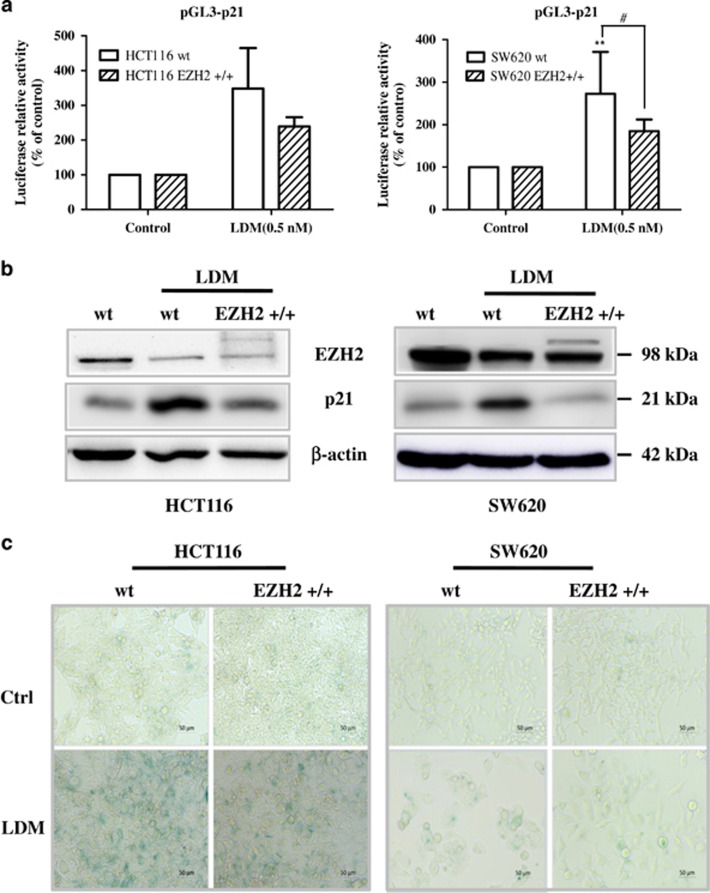
EZH2 overexpression reversed the induction of p21 promoter activity and protein expression as well as senescence caused by LDM. (**a**) Wild-type (wt) and stable cells infected with EZH2-expressing lentivirus (EZH2 +/+) were transfected with pGL3-p21 and Renilla plasmids for 6 h and treated with 0.5 nM LDM for another 72 h. Luciferase activity was measured and normalized with respective control (**P<0.01 *versus* control; ^#^*P*<0.05, *n*=3 independent experiments). (**b**) The cells were treated with LDM as above, and then harvested and subjected to IB analysis using respective antibodies. (**c**) EZH2 overexpression decreased LDM-induced senescent phenotype in both cells

To check the direct regulation between p21 and EZH2, we performed chromatin immunoprecipitation (ChIP) assay using EZH2 or H3K27me3 antibody in the selected region of *p21* promoter as reported.^24^ In Figure 5d, enrichment of both EZH2 and H3K27me3 proteins in the *p21* promoter region were found to be reduced by LDM. Accordingly, the increase of *p21* promoter activity was observed (Figure 6a). Meanwhile, stable expression of EZH2 by virus infection (EZH2 +/+) decreased the induction of both p21 mRNA and protein levels after the drug treatment, as evidenced by the luciferase activity and IB analysis, respectively (Figures 6a and b). Consistently, LDM-caused senescence was reduced by overexpressed EZH2 level (Figure 6c). Thus, EZH2 is proved to negatively regulate p21 expression and mediates LDM-induced senescence.

### EZH2 overexpression suppresses DNA-damaging effect upon LDM exposure

**Figure 7:**
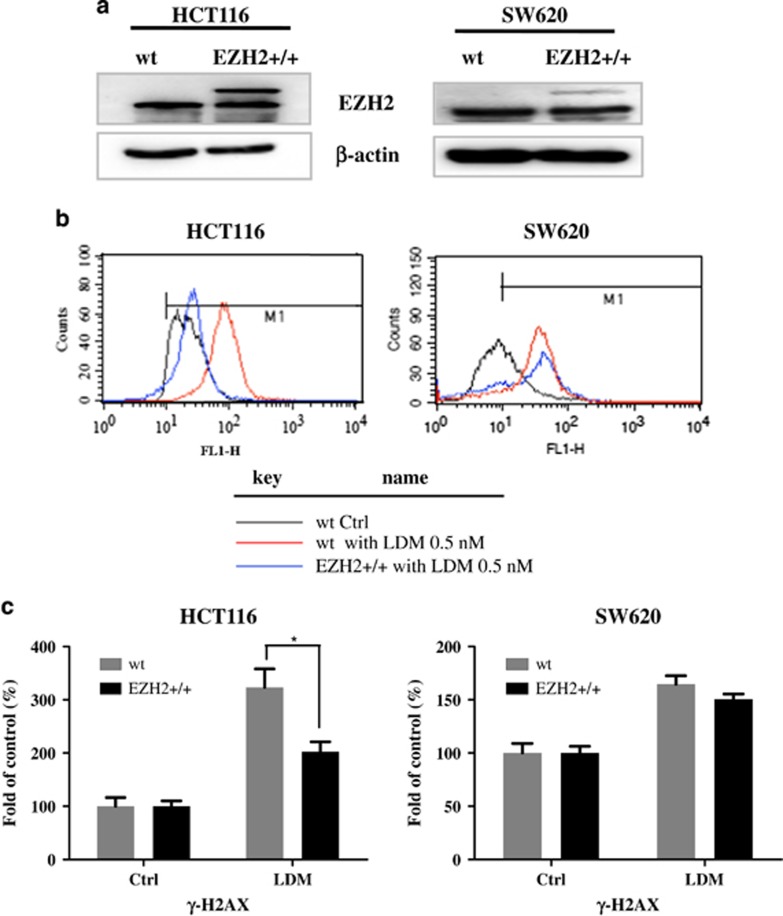
Overexpression of EZH2 decreased the DNA damage response caused by LDM. (**a**) Expression of EZH2 in wild-type and EZH2 +/+ cells as in Figure 6 were determined by IB analysis. (**b**) EZH2 overexpression decreased LDM-induced *γ*-H2AX level in both cell lines. Wild-type and EZH2 +/+ cell lines were seeded in six-well plates, and treated with LDM at 0.5 nM for 72 h, and then *γ*-H2AX level was analyzed by FACS as described in Materials and methods section. (**c**) Quantitation of the FACS analysis of three independent experiments (*P<0.05)

Previous studies have demonstrated that LDM's cytotoxicity results from its potent DNA-damaging effect, which is one of well-accepted inducers of cellular senescence,^21^ we next explored how EZH2 may affect the DNA-damaging efficacy of LDM. DNA-damaging effects were confirmed to exist time dependently after the drug treatment, as evidenced by the increase of *γ*-H2AX, a well-known marker of DNA damage, by IB analysis (Supplementary Figure S5). FACS analysis also demonstrated the increase of *γ*-H2AX by LDM, whereas this effect was suppressed in the cells with overexpressed EZH2 levels, especially in HCT116 cells (Figures 7a–c).

### LDM demonstrates potent *in vivo* antitumor activity

**Figure 8:**
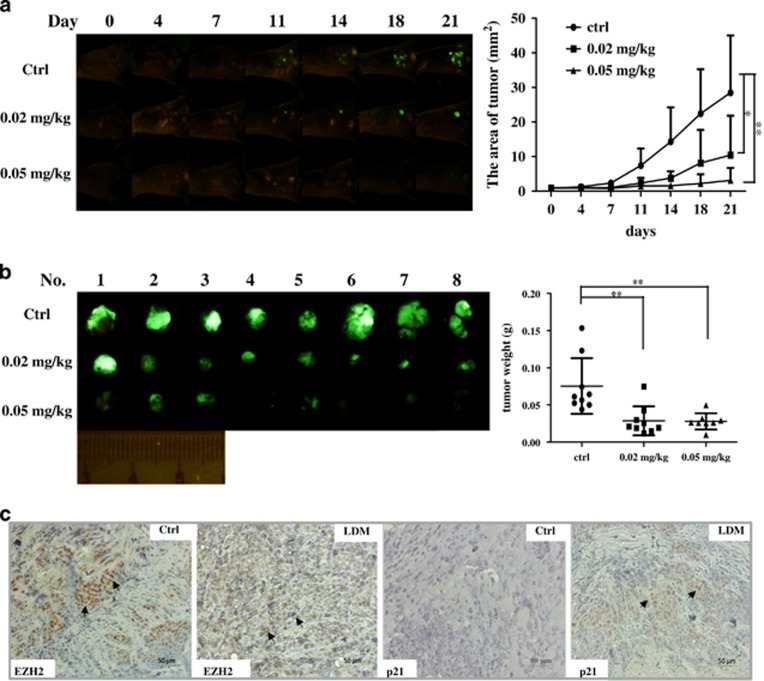
LDM inhibited the growth of orthotopic xenograft model of HCT116 colon cancer cells and affected EZH2 and p21 expression. (**a**) The whole-body imaging of tumor-bearing mice was monitored every 3 or 4 days from day 0 to day 21 (*n*=9) (*P<0.05, **P<0.01). (**b**) Mice were killed at the 21th day after treatment. Tumor tissues in each group were collected, photographed, and weighed (**P<0.01). (**c**) Expression of EZH2 and p21 expression in control and LDM treatment (0.05 mg/kg) group (representative of three slides in each group). The slides from the xenografted tumor tissues were stained by IHC with EZH2 and p21 antibodies. Scale bar represents 50 *μ*m for × 20 magnification. The arrows indicate positive staining area

We finally tested the *in vivo* antitumor effect of LDM in orthotopic xenograft of human colon cancer model established as described in Materials and methods section. Significant tumor growth inhibition by LDM was shown time dependently in Figure 8a, as monitored by whole body imaging of the tumor-bearing mice, whereas the agent also caused some loss of body weight (Figure 8a and Supplementary Figure S6). In Figure 8b, tumor volume and weight in three groups were shown, and LDM markedly inhibited the tumor growth upon 0.02 and 0.05 mg/kg treatment (both *P*<0.01 *versus* control). We further examined EZH2 and p21 expression in the xenografted tumor tissues by IHC analysis. In Figure 8c, the positive staining of EZH2 in control group was much stronger and more significant than that of LDM-treated group, which demonstrated a lighter and more diffuse staining pattern (as indicated by the arrows). Moreover, apparent positive staining was observed for p21 expression in the LDM-treated group compared with control group (Figure 8c). These findings showed that LDM exerts potent antitumor efficacy and depletes EZH2 expression *in vivo*.

## Discussion

Colon cancer has been the third leading cause of tumor-related death worldwide with high morbidity and mortality.^27, 28^ To restore the cellular senescence from permanent growth has been a valid tumor-suppressing strategy for cancer treatment. Cancer cell senescence refers to an irreversible growth arrest and may be induced by a variety of stresses.^21^ Here, we find that senescence becomes a vital antitumor mechanism induced by LDM in colon cancer cells, which is at least partially mediated by EZH2.

As a well-established anticancer drug, LDM exerts potent anticancer activities through the induction of apoptosis, mitotic cell death and cell cycle arrest etc., based on the dose range, treatment time and different cellular context.^7^ The cell cycle arrest caused by LDM in many types of cancer, including colon cancer, is mainly G2/M phase arrest because of decreased phosphorylation of Rb and increased phosphorylation of Chk1, Chk2 and Cdc25C etc.,^7, 18, 19^ and therefore constitutes one of major features of cellular senescence as well in this work (Figure 3). Moreover, although senescent phenotype, along with mitotic cell death, has been observed in BEL7402 liver cancer cells after transient LDM treatment for 2 h, the underlying mechanisms is far from being understood.^17, 29, 30^ In this work, we systematically characterized the role of senescence, as well as related mechanisms in human colon cancer for the drug.

EZH2 overexpression has been observed in many solid tumors, and correlates with poor prognosis.^3, 4, 5, 6^ We also confirmed higher EZH2 levels in colon cancer tissues compared with normal ones (Figure 1), indicating the protein is involved in the tumorigenesis of colon cancer. An interesting finding in this work is that LDM is relatively more potent in colon cancer cells carrying endogenously high EZH2 expression (Figure 2a), and can selectively deplete EZH2 instead of other histone lysine methyltransferases (Figure 4 and Supplementary Figure S3). As we know, this is the first report so far demonstrating the enediyne drug may directly regulate this important PRC2 member. This finding may point to a potential application of EZH2 as a target and biomarker to indicate the clinical efficacy of LDM and, possibly, the class of enediyne antibiotics, although further experiments have to be done to test this in other enediyne drugs. The regulation of EZH2 by LDM may occur at transcriptional or post-transcriptional level, as LDM-induced reduction of EZH2 fails to respond to proteasome inhibitor, and mRNA changes are simultaneously observed (Figure 4). Given that many previous reports have indicated the direct regulatory role of miRNAs for *EZH2* gene, including miR26 and miR101,^31, 32^ there is possibility that LDM may regulate these miRNAs to indirectly affect *EZH2* gene expression.

The role of EZH2 in cellular senescence has been demonstrated in some studies. For example, EZH2 levels are decreased during the late passage of primary MEFs cells, and knockdown of EZH2 induces premature senescence of MEFs, as well as human progenitor cells, partly due to loss of H3K27 trimethylation at the *Ink4a (p16)/Arf (p19)* locus.^14, 33^ Moreover, inhibition of EZH2 expression by siRNA or adriamycin causes senescent phenotypes in melanoma and gastric cancer cells, respectively, by inducing p21 and/or p16 activation.^16, 34^ Our previous work also proved the senescence-inducing ability of an EZH2 inhibitor DZNep in HCT116 cells.^35^ Although p16 induction is not observed, p21 is indeed regulated by EZH2 and contributes substantially to senescence induction after DZNep or LDM treatment. Meanwhile, the p21 induction by LDM occurs independently of p53. Consistently, epigenetic regulation of p21 by EZH2 regardless of p53 status is reported in other cancer types during senescence induction.^23, 34^ Furthermore, given LDM-caused p21 induction could not be totally reduced by EZH2 overexpression in HCT116 cells (Figure 6b), some p53-dependent regulation of p21 may not be excluded in the cells harboring wild-type p53.^36^

Notably, EZH2 and DNA methyltransferases (DNMTs) are reported to be interacting with and regulated by each other to repress target genes expression including the CDK inhibitors.^37, 38^ Therefore, despite EZH2, there is possibility that DNMTs such as DNMT1 may also be affected by LDM. We indeed detected a reduction of DNMT1 protein expression by LDM (data not shown). However, given that p16 is undetectable here, which is more tightly regulated by DNA methylation status and may easily be induced upon DNMT1 inhibition,^38, 39^ DNMT1 does not seem to be a major target for this drug. Further research is definitely needed to thoroughly understand this.

Apart from the epigenomic perturbations,^40^ senescence may be caused by the DNA-damaging effect of LDM. Our data showed EZH2 overexpression could decrease LDM-induced *γ*-H2AX, indicating EZH2 may be involved in the regulation DNA damage response with yet unknown mechanism. Although PRC2 is indicated to be recruited to the DNA damage sites in a poly(ADP-ribose) polymerase-dependent way and is involved in DNA damage repair,^41^ evidences about how EZH2 regulates DNA damage repair seem to be complicated and inconsistent. Some reports indicate EZH2 knockdown may affect DNA damage response by abrogating cell cycle checkpoints and reducing DNA double-strand break repair, therefore sensitizing the cells to cancer therapy,^41, 42^ whereas others show depletion of EZH2 results in enhanced DNA damage repair because of elevated expression of DNA damage repair genes.^43, 44, 45^ Apparently, more work has to be done to prove the regulatory role of EZH2 in LDM-induced DNA damage responses.

Taken together, our work provides first demonstration that EZH2 may mediate, at least partially, the senescence-inducing effects of LDM by regulating p21 expression and DNA-damaging efficacy. Thus, EZH2 may serve as a potential target and biomarker to indicate the clinical efficacy of the enediyne antitumor drug.

## Materials and Methods

### Reagents and cell lines

LDM was obtained from our institute and stocked in DMSO (Sigma-Aldrich, Saint Louis, MO, USA) at 50 mM. Annexin V-propidium iodide (PI) staining kit (FXP022-100) was purchased from Beijing 4A Biotech Co., Ltd, Beijing, China. Unless otherwise stated, all other chemicals were obtained from Beijing Chemical Works (Beijing, China). Antibodies for EED, p53, p16 and *β*-actin were purchased from Sigma-Aldrich, whereas other antibodies were from Cell Signaling Technology (Beverly, MA, USA). HCT116 cell line was obtained from China Infrastructure of Cell Line Resources in the School of Basic Medicine in Peking Union Medical College (Beijing, China). HCT116 p53−/− cells were gifted from Professor Bert Volgestein at Johns Hopkins University School of Medicine, Baltimore, MD, USA. All other cell lines were from ATCC (Manassas, VA, USA). EZH2-expressing lentivirus construct (pLVX-CMV-EGFP-EZH2-PGK-Puro) and virus particles were provided by Sunbio Medical Biotechnology Co., Ltd (Shanghai, China).

### Cell culture

HT29 cell line was cultured in Ham's F12 medium (Hyclone, Logan, UT, USA), whereas all other cell lines were maintained in DMEM (Hyclone), supplemented with 10% (*v/v*) fetal bovine serum (FBS, Life Technologies, Carlsbad, CA, USA), 100 U/ml penicillin G (Amresco, Solon, OH, USA) and 100 mg/ml streptomycin (Amresco). All the cells were cultured in a humidified atmosphere of 5% CO_2_ at 37 ^o^C. Unless otherwise stated, the concentration for LDM treatment throughout this work was 0.5 nM.

### IHC analysis of human tumor tissue array

IHC was performed on human colon cancer tissue microarrays containing 90 pairs of colon adenocarcinoma with adjacent normal tissues to compare the *in situ* expression of EZH2 by Shanghai Biochip (Shanghai, China). The tissue array sections of 4 *μ*m were dehydrated and subjected to peroxidase blocking, followed by incubation with EZH2 antibody (1 : 1000, #5246, Cell Signaling Technology) at room temperature for 30 min on the DAKO Autostainer (Dakocytomation, Carpinteria, CA, USA) using the Dako Cytomation EnVision+ System-HRP (DAB) detection kit. The sections were then counterstained with hematoxylin before observation. The staining intensity was classified into five groups with increasing staining intensity from marginal (±) to the strongest (++++) as described before.^46^ The fresh human colon tumor samples for IB were collected from Zhongshan Hospital in Shanghai, China, with written informed consent obtained from all patients.

### Cellular viability measurement

Cellular viability was performed with MTT (Amresco) assay. The cells were seeded at 5000 cells per well in 96-well plates for 24 h before treatment with LDM as indicated, and then incubated in MTT solution for 4 h at 37 °C. MTT solution was then carefully removed and replaced by 150 *μ*l DMSO to dissolve formazan crystals, followed by measurement with a microplate reader (Bio-Rad, Hercules, CA, USA). IC_50_ value and appropriate curve fit for each cell line was plotted with Graphpad Prism 5 (La Jolla, CA, USA).

### Cell cycle and apoptosis analysis

For cell cycle analysis, the cells were treated as indicated and harvested and fixed in 70% ethanol at −20 °C overnight, followed by staining with PI for 1 h after treatment with RNase at 37 °C for 30 min in the darkness. The stained cells were analyzed for DNA content by FACS analysis (BD Bioscience, San Jose, CA, USA). Apoptosis assay was performed using the Annexin V Alexa Fluor 488/PI apoptosis detection kit according to the manufacturer's instructions.

### Colony formation and cell growth curve assay

The cell lines were seeded at a density of 500 cells per well into 35 mm dishes and treated as indicated for 2 weeks. The colonies were fixed in 4% paraformaldehyde for 15 min and then washed with PBS followed by staining with hematoxylin. For the growth curve assay, the cells were seeded in triplicate into 24-well plate with 10^4^ cells per well for 24 h before drug treatment for the indicated time, and the cellular numbers were counted with Countess Cell Counter (Life Technologies).

### SA-*β*-gal staining

SA-*β*-gal staining was performed using the SA-*β*-gal Staining Kit (C0602, Beyotime Biotechnology, Jiangsu, China). Briefly, the cells were seeded at 3 × 10^5^ cells per well in six-well plates for 24 h, and then treated with LDM as indicated. The cells were then washed with PBS and fixed with 4% paraformaldehyde in PBS for 15 min at room temperature. The cells were then incubated overnight at 37 °C with the working solution containing 1 mg/ml X-gal in the kit, and senescence was identified as positive in the dark blue-staining cells observed. At least 200 cells were counted for each group in over three random fields to determine the percentage of SA-*β*-gal-positive cells.

### IB analysis

The cells were seeded at 3 × 10^5^ cells per well in six-well plates for 24 h, and then treated with LDM as indicated. The cells were washed with iced PBS and lysed with lysis buffer containing protease inhibitors cocktail (Cell Signaling Technology) plus 1 mM PMSF. Protein concentration was determined by Bradford assay. In all, 50 *μ*g of protein extracts were loaded and resolved by SDS-polyacrylamide gel electrophoresis, and transferred to PVDF membrane (Millipore Corporation, Billerica, MA, USA). The membranes were blocked with 5% nonfat milk PBS-T buffer at room temperature for 1 h, and then incubated for 2 h with primary antibodies at 1 : 1000 dilutions except for *β*-actin (1 : 5000). The membranes were then incubated for 1 h with an appropriate horseradish peroxidase-linked secondary antibody (Santa Cruz Biotechnology, Santa Cruz, CA, USA), and electrochemiluminescence was performed with ChemiImager 5500 imaging system (Alpha Innotech Corporation, San Leandro, CA, USA). Intensity values of representative blots were determined with ImageJ software (National Institutes of Health, Bethesda, MD, USA) and normalized to the intensity of respective loading control *β*-actin in each lane.

### Real-time PCR

RNA was extracted with TRIZOL reagent (Life Technologies), and then reversely transcribed using PrimeScript RT Master Mix (TaKaRa, Shiga, Japan). Quantitative real-time PCR was performed using a CFX96 Real-Time PCR Detection System (Bio-Rad). FastStart Universal SYBR Green Master (Roche, Basel, Switzerland) was used and fold change was calculated using the ΔΔCt method of relative quantification. Reactions were set for 30 cycles of denaturation at 94 °C for 30 s, annealing at 60 °C for 30 s and elongation at 72 °C for 1 min. The primers used were listed as follows: *EZH2* (forward primer, 5′-AATCAGAGTACATGCGACTGAGA-3′ reverse primer, 5′-GCTGTATCCTTCGCTGTTTCC-3′); *SUV39H1* (forward primer, 5′-CCTGCCCTCGGTATCTCTAAG-3′ reverse primer, 5′-ATATCCACGCCATTTCACCAG-3′); *EZH1* (forward primer, 5′-GAGTTGGTCGATGCCCTGAAT-3′ reverse primer, 5′-AGCATGTCGCTTTCTCTTTCTT-3′); *G9a* (forward primer, 5′-TCCAATGACACATCTTCGCTG-3′ reverse primer, 5′-CTGATGCGGTCAATCTTGGG-3′); GAPDH (forward primer, 5′-CATGAGAAGTATGACAACAGCCT-3′ reverse primer, 5′-AGTCCTTCCACGATACCAAAGT-3′).

### Gene knockdown and overexpression

The silencing of genes using 10 nM siRNAs (synthesized by RiboBio Co., Ltd, Guangzhou, China) was performed with Lipofectamine^TM^ RNAiMAX (Life Technologies) according to the manufacturer's recommendations. The siRNA sense sequences targeting *EZH2* were 5′-GACUCUGAAUGCAGUUGCU-3′ (1#), 5′-GCAAAUUCUCGGUGUCAAA-3′ (2#) and 5′-GAGGGAAAGUGUAUGAUAA-3′ (3#). The siRNA sense sequences targeting *p21* were 5′-CUUCGACUUUGUCACCGAG-3′ (1#), 5′-GACCAUGUGGACCUGUCAC-3′ (2#) and 5′-GGUCAGCGGUGAGCCAGAA-3′ (3#).

For the establishment of stable cell lines with high EZH2 expression, HCT116 or SW620 cells were infected with EZH2-expressing lentivirus particles (10–20 *μ*l with 4 *μ*g/ml polybrene) in 10 cm dishes and selected in the culture medium containing 5 *μ*g/ml puromycin.

### ChIP assay

The ChIP assay was performed using an Assay Kit (Merck Millipore, Darmstadt, Germany) following the manufacturer's instructions. Briefly, 1 × 10^7^ SW620 cells were cross-linked with 1% formaldehyde for 10 min at 37 °C, and then quenched with 0.125 M glycine. The chromatin was sonicated in the cold lysis buffer. Soluble chromatin was immunoprecipitated with Anti-EZH2 (#5246, Cell Signaling Technology) or H3K27me3 (#9756 Cell Signaling Technology). Normal rabbit IgG was used as control. DNA–protein immune complexes were eluted and reverse cross-linked, and DNA was then purified by phenol/chloroform extraction and ethanol precipitation. The amplification of DNA fragments was carried out using FastStart Universal SYBR Green Master. The presence of *p21* promoter domain in immunoprecipitated DNA was identified using the primers as described before.^24^ Reactions were set for denaturation at 94 °C for 1 min and annealing at 59 °C for 1 min, followed by elongation at 68 °C for 2 min. The amplified products were observed after 30 cycles and fold change was calculated using the ΔΔCt method of relative quantification. The ratio of a specific DNA fragment in each immunoprecipitate to that in input DNA was normalized.

### Dual-luciferase assay

HCT116 (EZH2 wt or EZH2 +/+) and SW620 cells (EZH2 wt or EZH2 +/+) were seeded in 96-well plates at 1 × 10^4^ cells per well for 24 h before transfection with the pGL3-p21-luc plasmid^47^ (100 ng per well) and Renilla (5 ng per well) in triplicate. After transfection for 6 h, the cells were treated with LDM for 72 h, and luciferase activity was measured using the Dual-luciferase Reporter Assay System kit (Promega, Madison, WI, USA) according to the manufacturer's instructions. Reporter firefly luciferase activity was normalized to Renilla luciferase activity.

### DNA damage response assayed by *γ*-H2AX level

The cells were harvested and fixed in 70% ethanol at 4 °C for 12–16 h, and then washed twice in PBS. Blocking was done by incubating with 10% (v/v) FCS for 1 h at room temperature. After washing in PBS, the cells were collected and treated with phospho-histone H2AX (Ser139) (*γ*-H2AX) antibody (#2577, Cell Signaling Technology) for 2 h at room temperature, followed by incubation with FITC-labeled goat anti-rabbit IgG (ZF-0311, ZSGB-BIO) for 1 h. The stained cells were washed and analyzed by FACS.

### Effect of LDM in xenograft tumor animal models

BALB/c mice (all 6-week-old females) were purchased from Hua Fu Kang (HFK) Bioscience Co., Ltd (Beijing, China). All the mice were maintained in a pathogen-free animal facility for at least 1 week before the experiment. The animal study was approved by the Institute's Animal Care and Use Committee. The antitumor efficacy of LDM was assessed in an orthotopic murine colon carcinoma model as described before.^48^ Briefly, HCT116-GFP cancer tissue that originated from subcutaneous tumor of the same species of nude mice was harvested and cut into small pieces of around 1 mm^3^. The small tumor tissue fragments were then inoculated into the incision site of the mucosa of cecum of each mouse through aseptic operation. After 72 h, the tumor-bearing mice were randomized into three groups (*n*=9 per group) and administrated with PBS or different doses of LDM (0.02 and 0.05 mg/kg) by tail vein injection once a week for two consecutive weeks. The whole-body imaging of tumor-bearing mice was monitored every 3 or 4 days from day 0 to day 21 by the FluorVivo Model 100 (I NDEC BioSystems, Santa Clara, CA, USA). All the mice were monitored daily for their general health status and weighed. The tumor growth curve was drawn accordingly. The mice were killed after 3 weeks and the tumor tissues were collected, photographed and weighed. IHC staining with EZH2 or p21 antibody incubation (1 : 100) was performed using the tissue sections derived from the xenografted tumor in a similar way as described above for the human tissue array.

### Statistical analysis

Statistical analysis was performed using Student's *t*-test (between two groups) or one-way ANOVA analysis (within-multiple groups) for data comparisons. In the case of IB analysis, one representative result from at least three experiments was shown. Statistical analysis were performed using SPSS 13.0(SPSS, Chicago, IL, USA), and differences between the groups were identified as statistically significant at three levels: *P*<0.05, *P*<0.01, and *P*<0.001.
