# Supplementary material for: EZH2 mediates lidamycin-induced cellular senescence through regulating p21 expression in human colon cancer cells
Source: Cell Death Dis. 2016 Nov 24;7(11):e2486–. doi: 10.1038/cddis.2016.383 (PMC5260875; doi:10.1038/cddis.2016.383)
Supplement: Supplementary Information [file cddis2016383x1.ppt]

## Slide 1
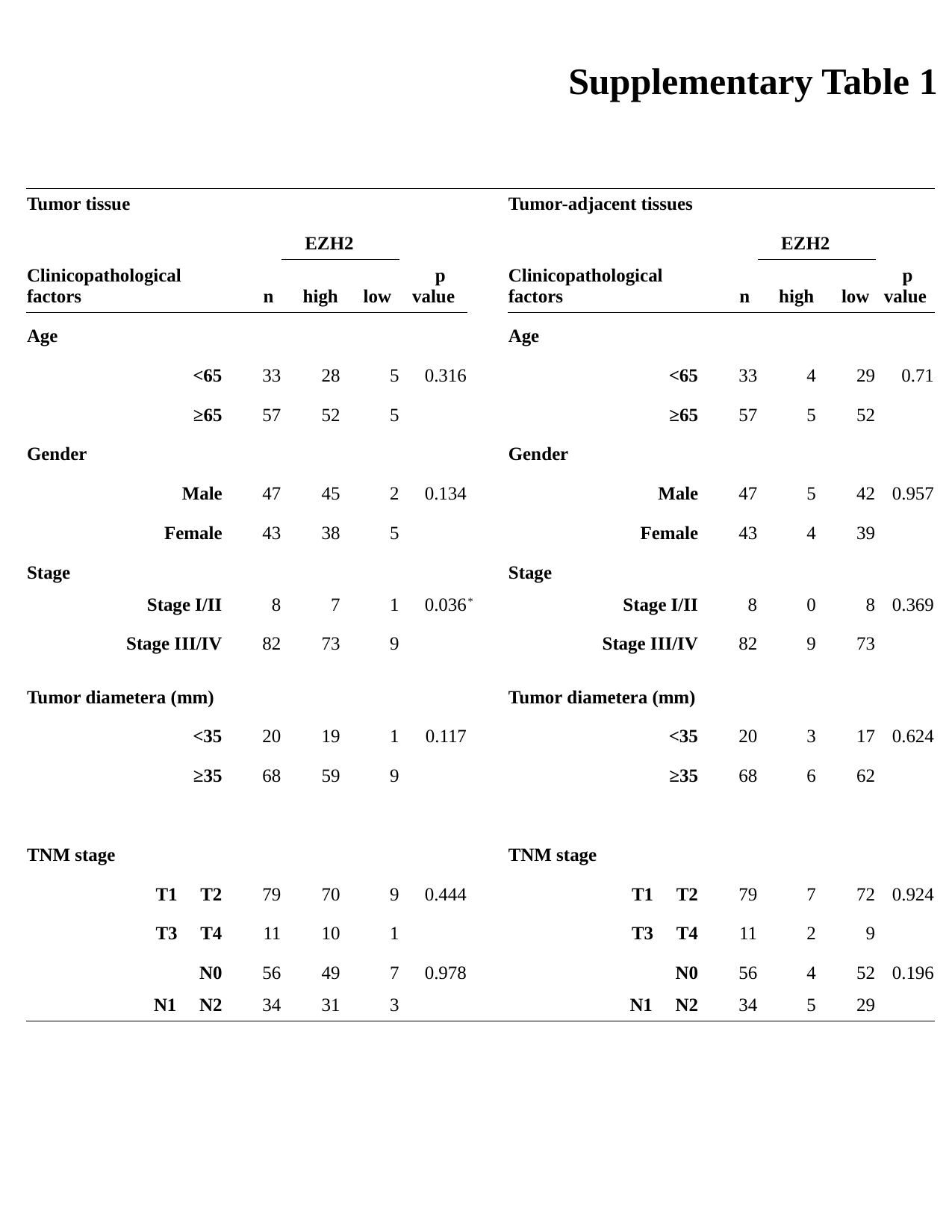

Supplementary Table 1
| | | | | | | | | | | |
| --- | --- | --- | --- | --- | --- | --- | --- | --- | --- | --- |
| Tumor tissue | | | | | | Tumor-adjacent tissues | | | | |
| | | EZH2 | | | | | | EZH2 | | |
| Clinicopathological factors | n | high | low | p value | | Clinicopathological factors | n | high | low | p value |
| Age | | | | | | Age | | | | |
| <65 | 33 | 28 | 5 | 0.316 | | <65 | 33 | 4 | 29 | 0.71 |
| ≥65 | 57 | 52 | 5 | | | ≥65 | 57 | 5 | 52 | |
| Gender | | | | | | Gender | | | | |
| Male | 47 | 45 | 2 | 0.134 | | Male | 47 | 5 | 42 | 0.957 |
| Female | 43 | 38 | 5 | | | Female | 43 | 4 | 39 | |
| Stage | | | | | | Stage | | | | |
| Stage I/II | 8 | 7 | 1 | 0.036 | \* | Stage I/II | 8 | 0 | 8 | 0.369 |
| Stage III/IV | 82 | 73 | 9 | | | Stage III/IV | 82 | 9 | 73 | |
| Tumor diametera (mm) | | | | | | Tumor diametera (mm) | | | | |
| <35 | 20 | 19 | 1 | 0.117 | | <35 | 20 | 3 | 17 | 0.624 |
| ≥35 | 68 | 59 | 9 | | | ≥35 | 68 | 6 | 62 | |
| | | | | | | | | | | |
| TNM stage | | | | | | TNM stage | | | | |
| T1，T2 | 79 | 70 | 9 | 0.444 | | T1，T2 | 79 | 7 | 72 | 0.924 |
| T3，T4 | 11 | 10 | 1 | | | T3，T4 | 11 | 2 | 9 | |
| N0 | 56 | 49 | 7 | 0.978 | | N0 | 56 | 4 | 52 | 0.196 |
| N1，N2 | 34 | 31 | 3 | | | N1，N2 | 34 | 5 | 29 | |
| | | | | | | | | | | |

## Slide 2
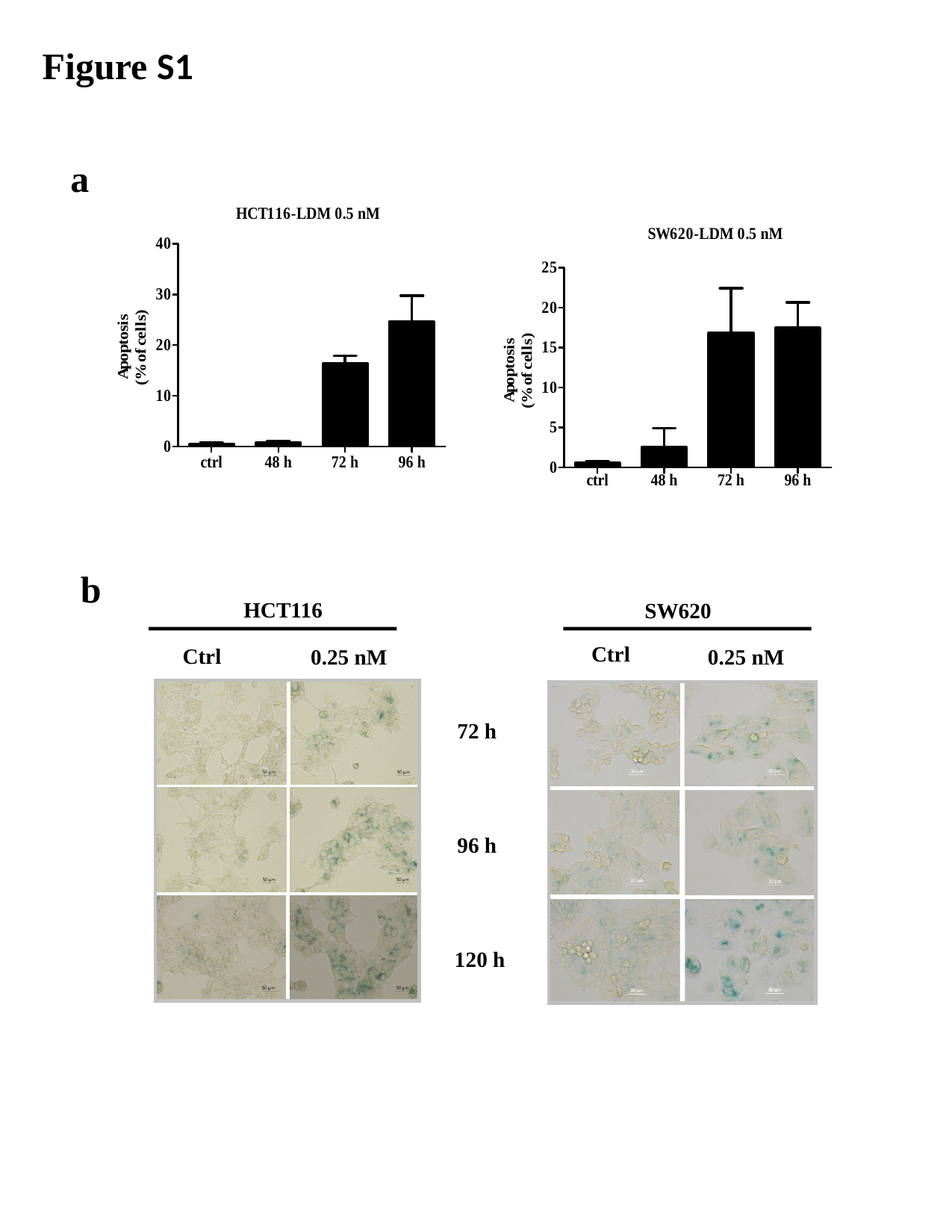

Figure S1
a
b
HCT116
SW620
Ctrl
Ctrl
0.25 nM
0.25 nM
72 h
96 h
120 h

## Slide 3
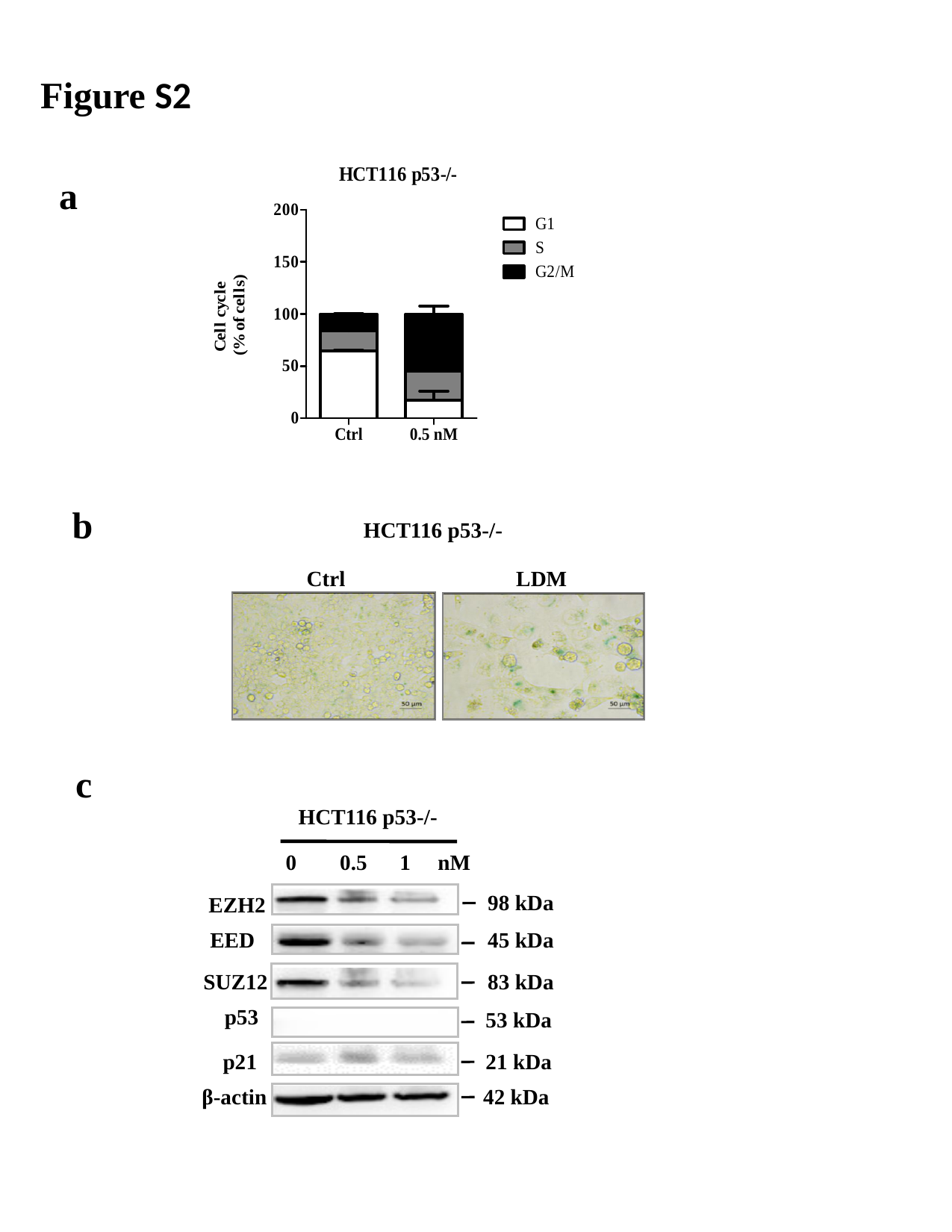

Figure S2
a
b
HCT116 p53-/-
Ctrl
LDM
c
HCT116 p53-/-
0 0.5 1 nM
EZH2
EED
SUZ12
p53
p21
β-actin
98 kDa
45 kDa
83 kDa
53 kDa
21 kDa
42 kDa

## Slide 4
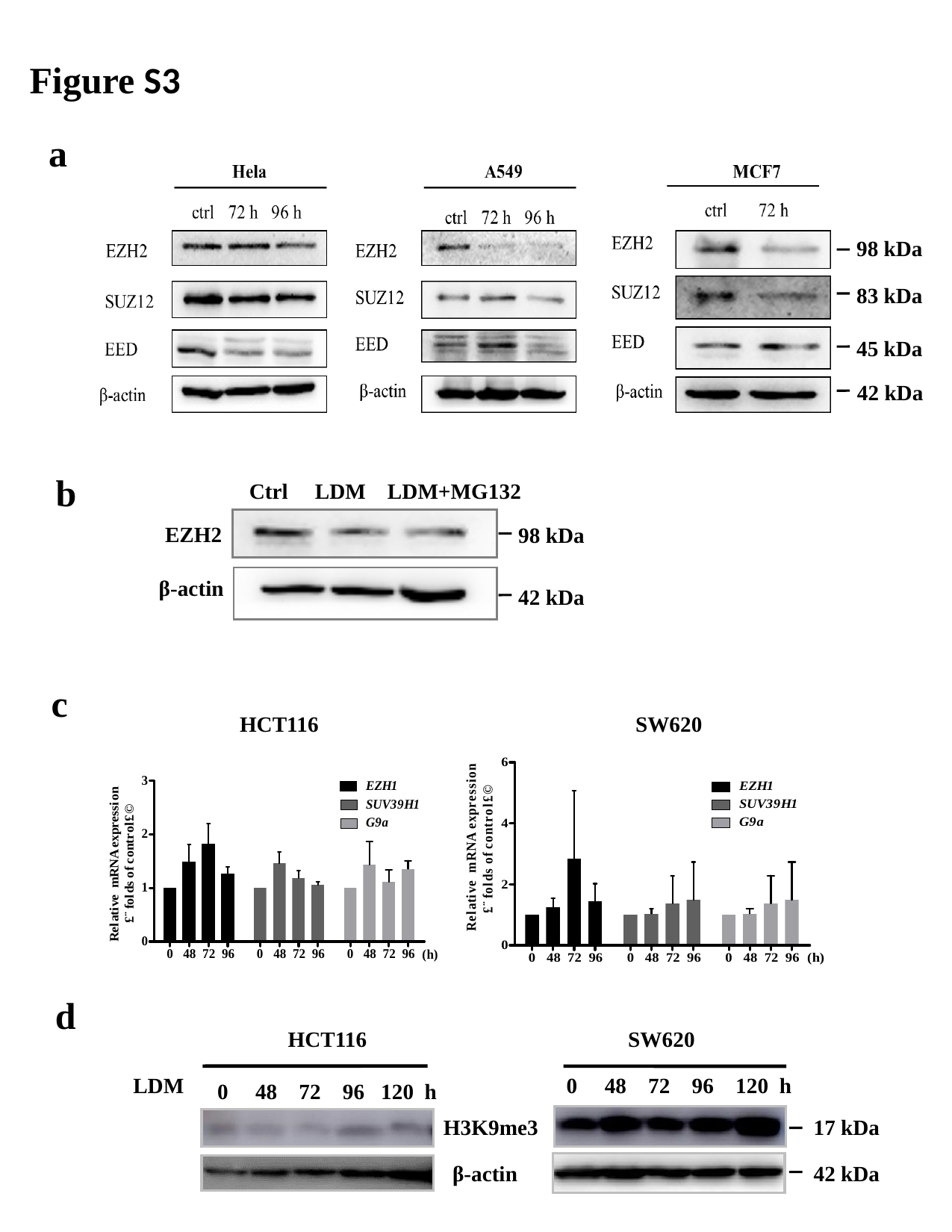

Figure S3
a
98 kDa
83 kDa
45 kDa
42 kDa
b
Ctrl LDM LDM+MG132
EZH2
β-actin
98 kDa
42 kDa
c
HCT116
SW620
d
HCT116
SW620
LDM
0 48 72 96 120 h
0 48 72 96 120 h
H3K9me3
17 kDa
β-actin
42 kDa

## Slide 5
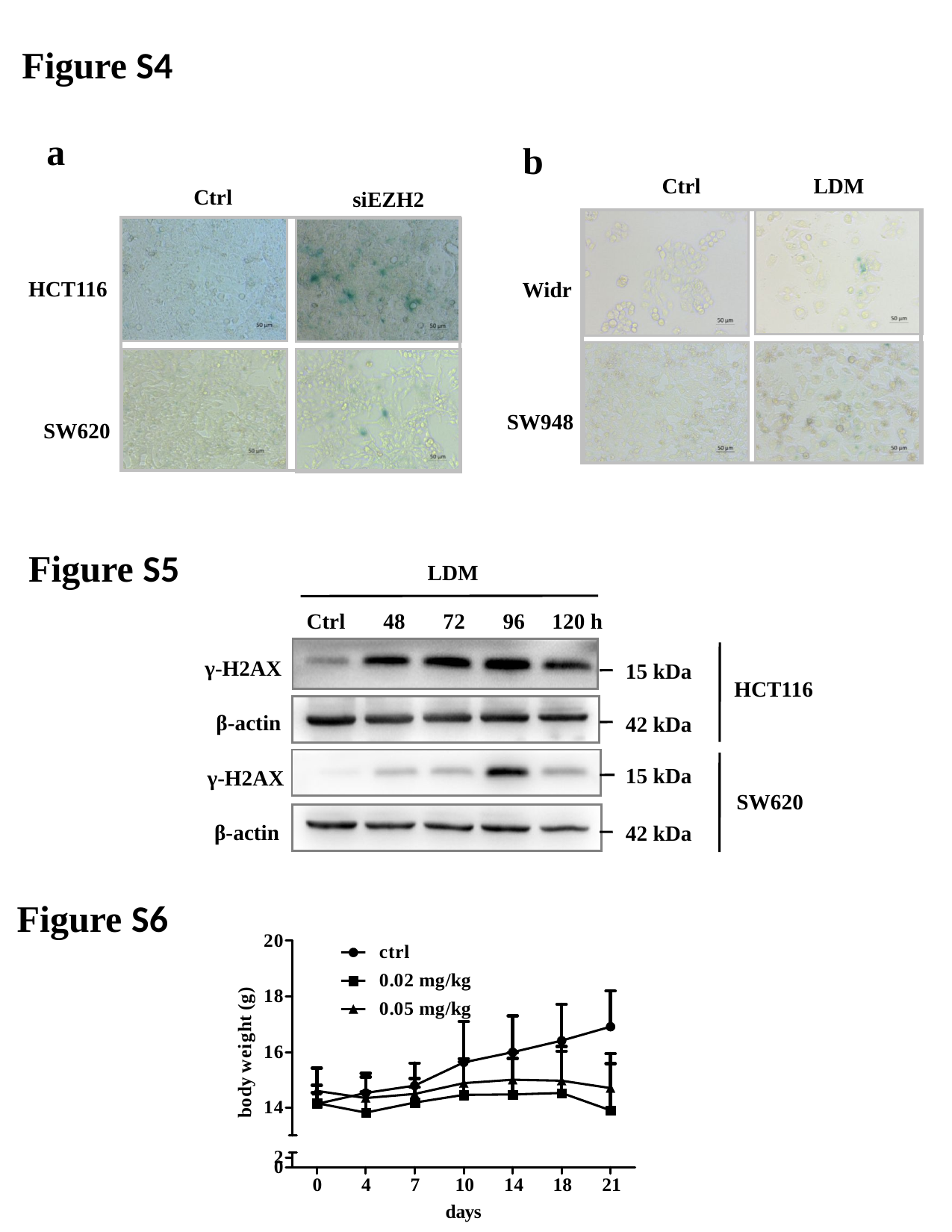

Figure S4
a
b
Ctrl
LDM
Ctrl
siEZH2
HCT116
Widr
SW948
SW620
Figure S5
LDM
Ctrl 48 72 96 120 h
γ-H2AX
β-actin
HCT116
γ-H2AX
β-actin
SW620
15 kDa
42 kDa
15 kDa
42 kDa
Figure S6
