# Supplementary material for: EZH2 mediates lidamycin-induced cellular senescence through regulating p21 expression in human colon cancer cells
Source: Cell Death Dis. 2016 Nov 24;7(11):e2486–. doi: 10.1038/cddis.2016.383 (PMC5260875; doi:10.1038/cddis.2016.383)
Supplement: Supplementary Figure Legends [file cddis2016383x2.doc]

**Supplementary figure & table legends**

Supplementary Table 1. Analysis of EZH2 protein expression in colon cancer according to clinical data.

Figure S1. (a) LDM induced apoptosis in HCT116 and SW620 cells for the indicated time (n=3 independent experiments). (b) LDM induced senescence in the colon cancer cells.

Figure S2. Effect of LDM on HCT116 p53-/- cells. (a) The cells were treated with 0.5 nM LDM for 72 h, and cell cycle distribution was analyzed by FACS. (b) LDM induced senescence in a p53-independent way. (c) LDM decreased EZH2 family member expression and increased p21 expression after 72 h treatment.

Figure S3. (a) LDM depleted EZH2 family member expression in other types of cancer cell lines. (b) The cells were treated with 0.5 nM LDM for 72 h in the absence or presence of 5 μM MG132, followed by IB analysis. (c) Gene expression of *EZH1*, *SUV39H1* and *G9a* after LDM treatment. The cells were treated with 0.5 nM LDM as indicated and subjected to qRT-PCR analysis (n=3 independent experiments). (d) Effect of LDM on the expression of H3K9me3 in both cell lines by IB analysis.

Figure S4. (a) Depletion of EZH2 by siRNA induced cell senescence in both HCT116 and SW620 cells. (b) Effect of LDM on Widr and SW948 cells. The cells were treated with LDM for 72 h, and cell senescence was analyzed with SA-β-Gal staining and photographed (20×). Scale bar: 50 μm.

Figure S5. LDM induced the expression of γ- H2AX (phospho-Ser139) in HCT116 and SW620 cells in a time-depended manner. The cells were seeded in 6-well plates, treated with or without LDM at 0.5 nM for the indicated time, then harvested and subjected to IB analysis.

Figure S6. Body weight of the animals measured during the treatment.
